# Multilineage polyclonal engraftment of Cal-1 gene-modified cells and in vivo selection after SHIV infection in a nonhuman primate model of AIDS

**DOI:** 10.1038/mtm.2016.7

**Published:** 2016-02-24

**Authors:** Christopher W. Peterson, Kevin G. Haworth, Bryan P. Burke, Patricia Polacino, Krystin K. Norman, Jennifer E. Adair, Shiu-Lok Hu, Jeffrey S. Bartlett, Geoff P. Symonds, Hans-Peter Kiem

**Affiliations:** 1 Clinical Research Division, Fred Hutchinson Cancer Research Center, Seattle, Washington, USA; 2 Calimmune, Inc., Los Angeles, California, USA; 3 Washington National Primate Research Center, Seattle, Washington, USA; 4 Department of Pharmaceutics, University of Washington, Seattle, Washington, USA; 5 Department of Medicine, University of Washington, Seattle, Washington, USA

## Abstract

We have focused on gene therapy approaches to induce functional cure/remission of HIV-1 infection. Here, we evaluated the safety and efficacy of the clinical grade anti-HIV lentiviral vector, Cal-1, in pigtailed macaques (*Macaca nemestrina*). Cal-1 animals exhibit robust levels of gene marking in myeloid and lymphoid lineages without measurable adverse events, suggesting that Cal-1 transduction and autologous transplantation of hematopoietic stem cells are safe, and lead to long-term, multilineage engraftment following myeloablative conditioning. Ex vivo, CD4+ cells from transplanted animals undergo positive selection in the presence of simian/human immunodeficiency virus (SHIV). In vivo, Cal-1 gene-marked cells are evident in the peripheral blood and in HIV-relevant tissue sites such as the gastrointestinal tract. Positive selection for gene-marked cells is observed in blood and tissues following SHIV challenge, leading to maintenance of peripheral blood CD4+ T-cell counts in a normal range. Analysis of Cal-1 lentivirus integration sites confirms polyclonal engraftment of gene-marked cells. Following infection, a polyclonal, SHIV-resistant clonal repertoire is established. These findings offer strong preclinical evidence for safety and efficacy of Cal-1, present a new method for tracking protected cells over the course of virus-mediated selective pressure in vivo, and reveal previously unobserved dynamics of virus-dependent T-cell selection.

## Introduction

HIV^+^ patients whose viremia is stably suppressed by combination antiretroviral therapy (cART) nevertheless exhibit increased morbidity and mortality relative to healthy controls, *e.g.*, due to HIV-dependent, ongoing immune activation.^1,2^ Recently, a number of strategies have emerged that seek to advance beyond the era of cART-dependent viral suppression, to achieve drug-free virus remission and/or functional cure.^3–5^ Among these, gene therapy-based approaches have been exemplified as being both safe in the clinical setting^3^ and capable of inducing remission.^6,7^

Stem cell gene therapy for HIV cure is predicated on the ability to introduce genetic protection against viral replication into hematopoietic stem cells (HSCs), which in turn pass this information onto infection-susceptible, stem cell-derived lymphoid and myeloid cell types, including T-cells, monocytes, and dendritic cells.^8^ A number of approaches have been evaluated *in vitro*, in animal models, and most recently, in clinical trials.^9^ These strategies can be broadly divided into viral vector-mediated delivery of antiviral transgenes, and nuclease-mediated targeting of virus-essential cellular genes or integrated proviral DNA. Viral vector-mediated strategies are advantageous in their ability to actively target multiple stages of the viral life cycle within a single multicistronic element.^10–12^

The efficacy of viral vector-mediated anti-HIV approaches has been demonstrated for several safety-engineered retroviral genera, including foamy virus^13^ and lentivirus,^14^ the latter being more extensively characterized in multiple experimental systems. Lentiviral strategies have been used to express short hairpin RNA,^15^ cell surface sequestration agents,^16^ and small peptide inhibitors of HIV entry.^17^ In addition to screening in multiple primary cell types *in vitro*,^18^ many approaches have undergone extensive evaluation in mouse models of HIV infection.^19,20^

We have tested the most promising lentiviral vector-based anti-HIV methods in nonhuman primates. We observed potent *ex vivo* inhibition of HIV-1 and SIV/HIV chimeric virus (simian/human immunodeficiency virus, SHIV) using foamy virus and lentiviral vectors expressing a small peptide inhibitor of virus fusion known as mC46, and shRNAs directed against essential virus and host genes.^21,22^ mC46 is derived from the C-terminal hydrophobic alpha helix region of HIV-1 gp41 and blocks virus entry by binding the N-terminal hydrophobic alpha helix of viral gp41 which, in turn, inhibits conformational changes required for virus fusion at the plasma membrane.^17^ Our previous work demonstrates that HSCs transduced with mC46-expressing lentiviral vectors and transplanted into autologous macaques give rise to lentivirus-marked progeny that efficiently resist subsequent SHIV challenge.^23^ Most recently, we have shown that this lentivirus-mediated autologous transplant strategy is safe and feasible in preclinical studies utilizing SHIV-infected animals that have been stably suppressed by cART.^24^

A critical parameter for successful gene therapy-mediated HIV cure strategies is the ability to generate a threshold number of infection-resistant cells. Below this threshold, protected cells would be overwhelmed by the number of infection-susceptible cells in the local microenvironment and could be rendered ineffective due to immune exhaustion and/or lost due to bystander-mediated cell death.^25,26^ Although the proportion of protected cells needed to provide a minimum protective effect against ongoing viral replication is unclear, we have previously employed a strategy to positively select for lentivirus-marked cells using the *in vivo* positive selection marker MGMT^P140K^.^27^ P140K chemoselection is safe and feasible in the clinical setting and has already shown efficacy in clinical trials in glioblastoma patients.^28,29^ In our previous studies, P140K chemoselection led to lentivirus marking levels of ~20% in peripheral blood cells of nonhuman primates prior to SHIV challenge. This level of protection was sufficient to induce marked improvements in the host immune response to SHIV infection.^23^

We are interested in the ability to transplant anti-HIV lentiviral vector-marked cells following conditioning of the hematopoietic compartment with chemotherapeutic agents such as busulfan or total body irradiation. We aim to generate sufficient quantities of protected cells to effectively resist ongoing viral replication and do so either in the presence or absence of P140K-mediated chemoselection. Here, we transplanted three pigtailed macaques (*Macaca nemestrina*) with a clinical-grade lentiviral vector expressing mC46 and a shRNA specific to human CCR5 and determined the level of engraftment that was achievable when using myeloablative conditioning without chemoselection. We subsequently challenged with SHIV, measured the extent of SHIV-mediated positive selection for lentivirus-marked cells, and assessed the safety of our lentiviral protection strategy.

## Results

### Study design

Previous results from our group demonstrated that CD4^+^ T-cells were protected following SHIV challenge in animals which had received autologous transplant with mC46 lentivirus-transduced HSCs, following MGMT^P140K^-dependent chemoselection.^23^ To broaden the potential clinical application of this antiviral strategy, we evaluated a clinical-grade mC46 vector, named Cal-1, which expresses mC46 under the control of the Ubiquitin C promoter, and a human CCR5 shRNA under the control of the RNA PolIII H1 promoter.^30^ Although the human CCR5 shRNA does not target macaque CCR5,^31^ we chose to evaluate the mC46-dependent properties of this vector in macaque transplant studies as a comparison to ongoing clinical trials that utilize Cal-1 in HIV^+^ patients.

**Figure 1:**
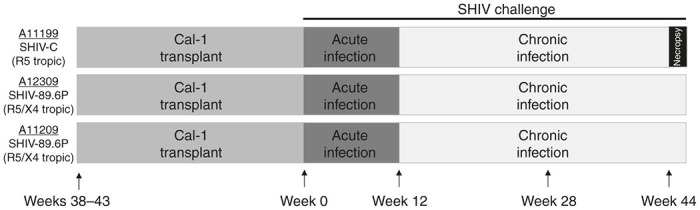
Experimental outline for Cal-1 transplant and SHIV challenge experiments. Three animals (ID A11199, A12309, and A11209) were transplanted with autologous CD34^+^ hematopoietic stem cells transduced with clinical-grade lentivirus expressing the fusion inhibitor C46 (“Cal-1”). Nine to eleven months following transplant, animals were challenged with CCR5-tropic env-SHIV (A11199; SHIV-C) or CCR5/CXCR4-tropic env-SHIV (A12309, A11209; SHIV-89.6P). A11199 was necropsied 10 months following challenge; A12309 and A11209 remain under study. SHIV, simian/human immunodeficiency virus.

An outline of our study is shown in Figure 1. Three animals (IDs A11199, A12309, and A11209) underwent autologous transplantation, including transduction of HSCs with Cal-1 essentially as previously described.^32^ Transductions were performed once, with a multiplicity of infection of 5, to more closely approximate the clinical transduction protocol. Hematopoietic recovery was monitored in peripheral blood following myeloablative conditioning and subsequent autologous cell transplantation, and the level of engraftment of gene-modified cells was determined over time. Ten to eleven months posttransplant, all three animals were challenged with SHIV via the intravenous route. Animal A11199 was challenged with 9500 TCID50 of the CCR5-tropic, SHIV1157-ipd3N4 (“SHIV-C”),^33,34^ while animals A12309 and A11209 were challenged with 50 MID50 of the dual-tropic SHIV89.6P.^35–37^ A11199 was monitored for 11 months, followed by end-of-study necropsy. A12309 and A11209 currently remain under study (Figure 1).

### Cal-1 autologous transplantation is safe, and gene-marked cells engraft *in vivo*

**Figure 2:**
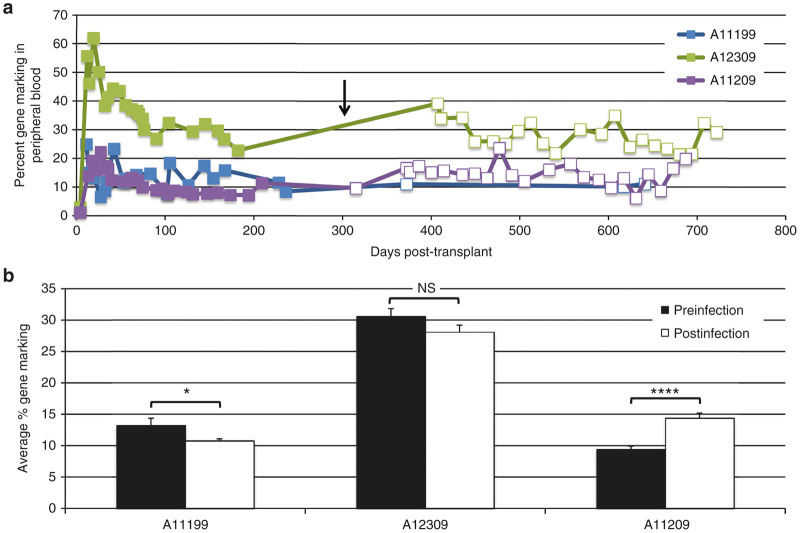
Gene marking in three pigtailed macaques following autologous transplant with Cal-1-transduced CD34^+^ cells. (**a**) Total lentiviral vector DNA was quantified by Taqman from animal ID A11199 (blue bars), A12309 (green bars), or A11209 (purple bars) following transplant and SHIV challenge. Arrow indicates SHIV challenge, and open squares indicate time points collected following SHIV challenge. (**b**) Average gene marking values pre- (black bars) and postinfection (white bars) for each animal. Preinfection values were collected following posttransplant stabilization of platelet counts. 100% gene marking represents 1 copy of vector provirus per cell, based on a standard curve constructed from a single-copy lentivirus-infected cell line. Error bars represent standard error of the mean for at least three independent time points. *P* values: NS: not significant; *P ≤ 0.05; ****P ≤ 0.0001. SHIV, simian/human immunodeficiency virus.

To ascertain whether transplant with Cal-1-transduced cells led to any gross impairment in hematopoietic reconstitution following autologous transplant, we monitored total white blood cell, platelet, neutrophil, and lymphocyte recovery in the three transplanted animals starting from infusion of transduced cells, through hematopoietic recovery, until SHIV challenge (Supplementary Figure S1). Recovery of each of these hematopoietic subsets was consistent with our past observations, suggesting that Cal-1 transduction does not impinge on hematopoietic reconstitution following a conditioning regimen consisting of 1,020 cGy total body irradiation.^27^ We next measured the *in vivo* gene marking in total leukocytes collected from each animal at longitudinal time points following transplant. Since our clinical vector lacked a fluorescent marker, gene marking was measured by genomic DNA taqman against Cal-1 lentiviral sequence. Shortly after transplant, animals A11199 and A11209 showed similar levels of peak gene marking (20–25%), while animal A12309 peaked at over 60% (Figure 2a). We defined steady-state gene marking based on stable recovery of platelet counts following total body irradiation (see Materials and Methods). Following a typical 63–67 days recovery period posttransplant, we observed pre-SHIV steady-state gene marking levels of 13.23 and 9.38% in A11199 and A11209, respectively, and 30.59% in A12309 (Figure 2b). Following SHIV challenge, gene marking levels in total leukocytes were similar to prechallenge levels (Figure 2a); a slight decrease in average gene marking was observed in animals A11199 and A12309, and an increase was seen in animal A11209 (Figure 2b). These data show that autologous transplantation utilizing Cal-1 lentiviral vectors is safe, and mediates stable gene marking in macaque peripheral blood (9–31%) following a single transplant using myeloablative conditioning and without methods of selection for gene-modified cells.

### Colony forming capacity and SHIV resistance of Cal-1 gene-marked cells *ex vivo*

**Figure 3:**
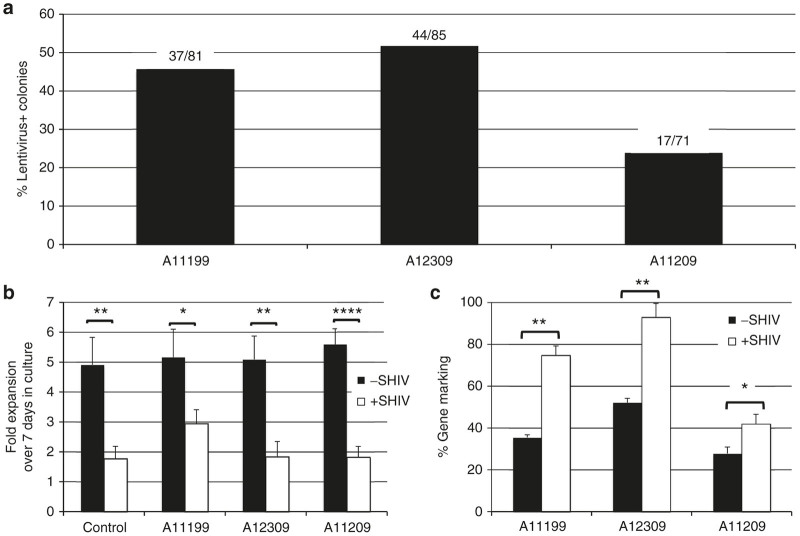
*Ex vivo* measurements of Cal-1 gene marking and protection against SHIV challenge. (**a**) Prior to infusion into the indicated animal, Cal-1 GLP-transduced CD34^+^ cells were plated for colony forming assays. Colonies were picked from a total of three plates and screened by PCR for lentiviral backbone and actin. Values over each bar represent the number of lentivirus-positive colonies (numerator) as a function of total actin-positive colonies (denominator) (**b, c**). CD4^+^ cells were collected from the indicated animals following transplant recovery, challenged ex vivo with SHIV-Ku1 at a multiplicity of infection (MOI) of 0.05, and followed over a 7-day time course. **b** Fold expansion of cells from one non-transplanted control, and the indicated Cal-1-transplanted animals. **c** Lentivirus gene marking was measured by Taqman. Data represent average and standard error of the mean for at least three replicate analyses from two independent experiments. *P* values: *P ≤ 0.05; **P ≤ 0.01; ****P ≤ 0.0001. SHIV, simian/human immunodeficiency virus.

A primary goal of this study was to directly compare the level of Cal-1 gene marking in transduced CD34^+^ HSCs and HSC-derived T-cells *in vivo* with results obtained in *ex vivo* studies. We first measured gene marking in the transplanted HSC product from each animal in colony forming assays. Cal-1 marking was detected in between 25 and 55% of assayed colonies from each of the three animals (Figure 3a). Animal A12309, which displayed the greatest transduction levels of CD34^+^ cells, also displayed the greatest level of gene marking in peripheral blood and tissue prior to infection (Figures 2, 5, 6). Our past results demonstrate a robust positive selection for mC46-modified cells *ex vivo* and *in vivo.*^22,23^ Therefore, we next evaluated the ability of isolated, SHIV-naive CD4^+^ cells from Cal-1-transplanted animals to resist infection with SHIV *ex vivo*. We used the CXCR4-tropic SHIV-Ku1 for these experiments, due to its ability to efficiently replicate in cultured primary pigtailed macaque cells.^22^ Consistent with our past results, SHIV-Ku1 significantly limited the expansion of stimulated CD4^+^ cells from transplanted animals, and an untransplanted control over a 7-day *ex vivo* culture period (Figure 3b). Because the majority of cells in these assays from transplanted animals were not gene marked (Figure 2a), and animal-to-animal variability exists in the ability of CD4^+^ cells to propagate in this assay (Figure 3b and data not shown), the variability in cell proliferation in transplanted animals was unsurprising. We therefore asked whether *ex vivo* SHIV challenge led to positive selection for Cal-1 gene-marked cells in these assays. We isolated total genomic DNA from the challenge cultures from Figure 3a and performed Taqman assays as described above. Cal-1 marking was increased approximately twofold in each of the three transplanted animals (Figure 3c). In summary, our *ex vivo* assays show that Cal-1 transduction efficiency of CD34^+^ cells correlates with gene marking levels in peripheral blood and tissues of transplanted animals measured prior to infection, that CD4^+^ cells from Cal-1-transplanted animals are resistant to *ex vivo* SHIV infection, and that gene-marked cells undergo virus-dependent positive selection.

### Healthy CD4^+^ T-cell counts in peripheral blood of transplanted, SHIV-infected animals

**Figure 4:**
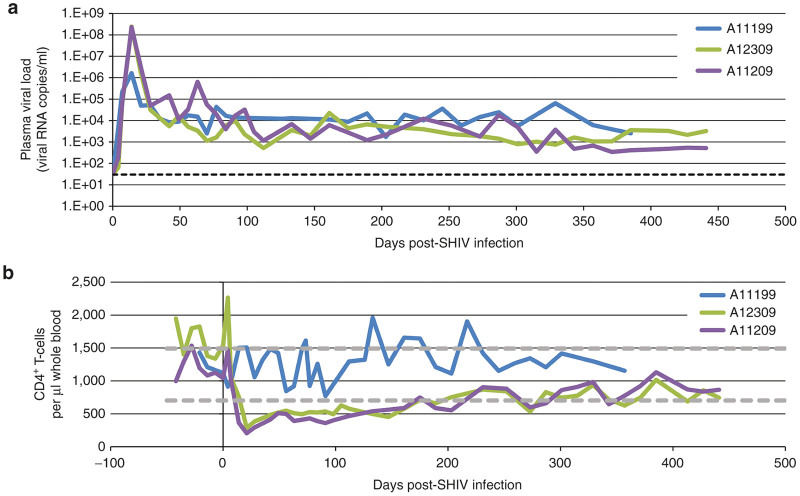
Plasma viral loads and CD4^+^ T-cell dynamics in Cal-1-transplanted animals following SHIV challenge. At 9–11 months after autologous transplant, animal ID A11199 was challenged with SHIV-1157ipd3N4 (“SHIV-C,” blue bars) and animals A12309 and A11209 were challenged with SHIV 89.6P (green and purple bars, respectively) by the intravenous route. (**a**) SHIV genomic RNA was measured by quantitative RT-PCR longitudinally in each animal. Dotted line represents 30 copies/ml, the limit of detection of the assay. (**b**) Longitudinal CD3^+^CD4^+^ T-cell measurements were made by flow cytometry. Dotted lines represent range of CD4^+^ T-cell counts from 26 healthy control animals. SHIV, simian/human immunodeficiency virus; RT-PCR, reverse transcription PCR.

We next challenged our transplanted animals with SHIV to evaluate whether plasma viral loads and peripheral blood CD4^+^ T-cell counts were comparable to historical untransplanted controls. Animal A11199 was challenged with the CCR5-tropic SHIV1157-ipd3N4 (“SHIV-C,” Figure 1). Compared with previously published controls using an identical challenge stock,^34^ plasma viral load in this animal was not significantly impacted by Cal-1 within the time frame monitored (Figure 4a). Findings from our group and others demonstrate that SHIV-C leads to depletion of peripheral blood CD4^+^ T-cell counts within 6 months of infection in approximately one-third of challenged animals, and progression to simian AIDS in 88–270 weeks.^34,38^ We did not observe either of these parameters in A11199 over ~44 weeks postchallenge (Figure 4b). To address the possibility that the lack of progression was due to the slower kinetics of pathogenesis of this CCR5-tropic SHIV, we challenged animals A12309 and A11209 with the CCR5 and CXCR4 dual-tropic SHIV 89.6P (Figure 1), which manifests more severe depletion of peripheral blood CD4^+^ T-cells and rapid progression to simian AIDS.^36,37^ At 13 weeks post infection, transplanted animals that were challenged with SHIV89.6P displayed greater than 1 log decrease in plasma viral loads (Figure 4a) relative to historical controls that were inoculated with the identical challenge stock.^35^ Plasma viral loads continued to slowly decline in these animals in the following months and were maintained predominately under 10,000 copies/ml. Furthermore, historical control animals exhibited low to undetectable CD4^+^ T-cell counts in peripheral blood in as little as 4 weeks following SHIV challenge; this severe and sustained course of pathogenesis led to simian AIDS and animals were euthanized between 38 and 62 weeks postchallenge. In stark contrast, Cal-1-transplanted animals displayed sustained increases in CD4^+^ T-cell counts over time following SHIV 89.6P challenge. We calculated a healthy CD4^+^ T-cell count range from multiple measurements from 26 healthy, SHIV-naive pigtailed macaques: Cal-1-transplanted, SHIV89.6P-challenged animals achieved counts comparable to healthy controls at 25 weeks postchallenge, and maintained these levels throughout the subsequent course of study. These animals remain healthy over 1 year post-challenge, whereas two-thirds of historical control animals had progressed to simian AIDS within 1 year. These findings suggest that Cal-1-transplanted animals are protected against SHIV-dependent immunodepletion and can stably maintain a reduced viral load *in vivo.*

### Subset-specific enrichment of Cal-1-marked cells following SHIV challenge

**Figure 5:**
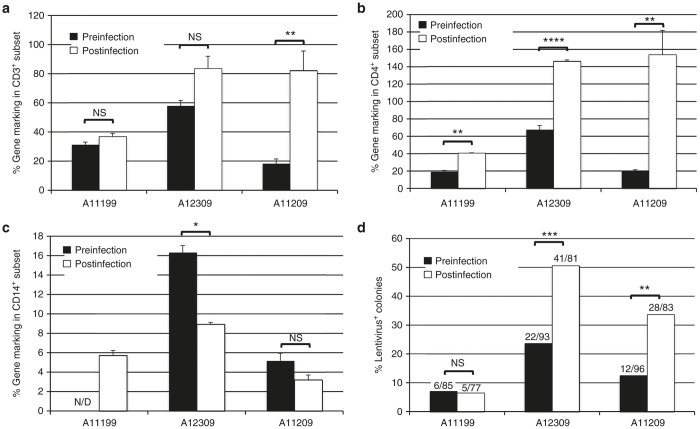
Gene marking in hematopoietic subsets following Cal-1 transplant and SHIV challenge. Before (200–300 days posttransplant; black bars) or after SHIV challenge (100 days postinfection, white bars), whole blood and bone marrow were collected from the indicated Cal-1-transplanted animal. Bead-based positive selection was used to isolate peripheral blood CD3^+^ (**a**), CD4^+^ (**b**), and CD14^+^ cells (**c**), followed by lentiviral gene marking measurement by Taqman. 100% gene marking represents an average of 1 copy of vector provirus per cell, based on a standard curve constructed from a single-copy lentivirus-infected cell line. Error bars represent standard error of the mean for 2–5 independent Taqman analyses of the same gDNA sample. (**d**) Bone marrow was hemolysed, and total leukocytes were plated for colony-forming assays. Shown is the percentage of PCR-screened colonies that were positive for actin and lentiviral backbone versus actin alone. Values over each bar represent the number of lentivirus-positive colonies (numerator), as a function of total actin-positive colonies (denominator). *P* values: NS: not significant; *P ≤ 0.05; **P ≤ 0.01; ***P ≤ 0.001; ****P ≤ 0.0001. SHIV, simian/human immunodeficiency virus.

Cal-1 gene marking in total leukocytes was not drastically altered in Cal-1-transplanted animals following SHIV challenge (Figure 2b). To examine SHIV-dependent changes in Cal-1 gene marking in hematopoietic subsets, we performed bead-based sorting experiments and colony forming assays. We Ficoll-separated peripheral blood from each animal before and after SHIV challenge, sorted CD3^+^, CD4^+^, and CD14^+^ subsets from the mononuclear cell layer, and prepared total genomic DNA from each subset for Cal-1 gene marking analysis by Taqman. We detected Cal-1 in all subsets measured, suggesting that transduced HSCs were capable of multilineage engraftment (Figure 5). Further, we observed positive selection for Cal-1 in CD3^+^ cells (Figure 5a), and more so in CD4^+^ cells (Figure 5b), but not in CD14^+^ myeloid cells (Figure 5c) following SHIV challenge. In parallel to these analyses, colony-forming assays were plated from total bone marrow white blood cells before and after SHIV challenge. Prior to SHIV challenge, marking ranged from 7 to 24%. Following SHIV challenge, colony marking was unchanged for animal A11199, but was increased between two to threefold for animals A12309 and A11209 (Figure 5d). These findings demonstrate that Cal-1 gene marking is multilineage, and that SHIV-dependent enrichment of Cal-1-marked cells is evident in HSC subsets and in SHIV-susceptible subsets; virus-dependent selection is most notable following challenge with the virulent dual-tropic strain 89.6P (Figure 4).

### Cal-1 gene-marked cells are detected in secondary lymphoid tissue

**Figure 6:**
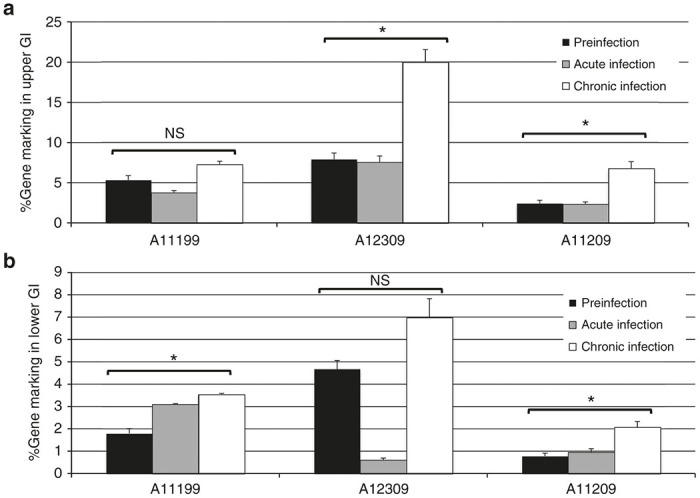
Gene marking in gastrointestinal biopsies from Cal-1-transplanted animals before and after SHIV challenge. Gastrointestinal biopsies were collected from the duodenum/jejunum (“Upper GI,” (**a**)) and colon (“Lower GI,” (**b**)) from the indicated animal at time points prior to SHIV challenge (“Preinfection”), 2 weeks post-SHIV challenge (“Acute infection”) or 10–11 weeks post-SHIV challenge (“Chronic infection”). Following isolation of single-cell suspensions from biopsy samples, gDNA was prepared, and lentiviral gene marking was assessed by Taqman. 100% gene marking represents 1 copy vector provirus per cell, based on a standard curve constructed from a single copy lentivirus-infected cell line. *P* values: NS: not significant; *P ≤ 0.05. SHIV, simian/human immunodeficiency virus.

The ability of mC46-protected hematopoietic cells to traffic to secondary lymphoid tissues such as gastrointestinal (GI) tract will be essential to effectively target viral reservoirs that are induced upon initiation of cART. Therefore, we conducted upper and lower GI biopsies from duodenum/jejunum and colon, respectively, from each of the three animals to detect Cal-1 gene marking at these sites. Biopsy pinches were collected immediately prior to SHIV challenge, and 2 weeks (“Acute Infection”) or 10–11 weeks (“Chronic Infection”) following challenge. Pinches were dissociated in the presence of collagenase and total genomic DNA was isolated for gene marking analysis by Taqman. Importantly, only a subset of the biopsied cell material was expected to be of hematopoietic origin; hence, we could not control for the variable proportion of HSC-derived cells in each sample. We detected Cal-1-marked cells in pre-SHIV upper (Figure 6a) and lower GI biopsies (Figure 6b) from all three animals. We observed an upward trend in marking in all three animals following SHIV challenge, except for acute infection samples from A11199 upper GI and A12309 lower GI; these could be due to sampling variability and/or depletion of hematopoietic cells in the gut during this phase of infection (Figure 6). However, the significant increase in gene marked cells between pre-SHIV and 10–11 week post-SHIV samples suggests that transplanted cells do home to the gut, and undergo SHIV-dependent positive selection, analogous to our observations in peripheral blood.

### Cal-1 gene-marked cells exhibit polyclonality both pre- and post-SHIV infection

**Figure 7:**
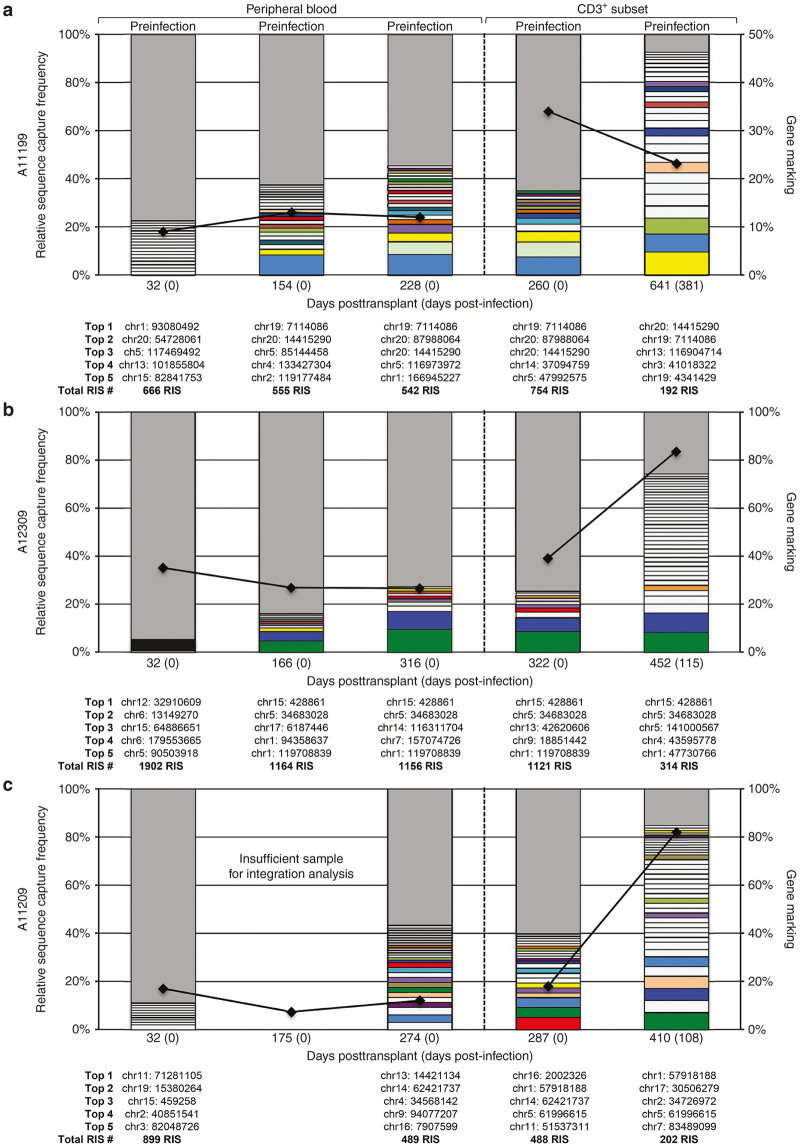
Integration of Cal-1 is polyclonal and persists following SHIV challenge. Retrovirus integration site (RIS) analysis was used to measure the total number of integration sites detected for animal IDs A11199 (**a**), A12309 (**b**), and A11209 (**c**) following transplant and SHIV challenge. Time points are indicated on the *x*-axis of each graph and correspond to days posttransplant or days postinfection in (parentheses). The first three columns represent peripheral blood samples while the two right columns represent CD3^+^ enriched fractions pre- and postinfection. All clones which represent >1% of sequences captured are shown as boxes with the remainder represented by a single grey box. Unique integration sites detected at multiple sampling times from each animal are color coded. Chromosomal locations of the top five sites are listed below each column in order of most frequent. Total RIS sites detected at each condition are shown underneath each chromosomal table. The corresponding gene marking for each sample is shown as a black line between samples and is plotted on the right *y*-axis. SHIV, simian/human immunodeficiency virus.

To ensure that Cal-1-transduction was not associated with expansion and potential oncogenic transformation of clonal subsets, we performed retrovirus integration site analysis as previously described.^28^ For each animal, we analyzed the clonal distribution of Cal-1-marked cells in total peripheral blood at ~1, 6, and 9 months posttransplant, prior to SHIV challenge (Figure 7). We observed a high level of polyclonality in all of these samples: greater than 450 unique clones were detected at each time point per animal. In no sample did we observe a single clone which represented >10% of the total clonal pool detected from that animal and time point, whereas the vast majority of clones represented <1% of integration sites detected. Numerous identical clones were detected from each animal over time, indicating the emergence of cell progeny from Cal-1-marked, long-term repopulating HSC’s. Over 1,100 unique clones were identified in animal ID A12309 at each time point preinfection, consistent with the higher gene marking observed in this animal (Figure 7b). Specific analysis of integration sites in proximity to genes previously linked to oncogenic transformation in clinical trials for X-linked Severe Combined Immunodeficiency (X-SCID),^39,40^ chronic granulomatous disease ^41^ and Wiskott–Alrdrich Syndrome (WAS)^42,43^ was performed (Supplementary Table S1). All three animals contained detectible clones with integration sites ranging from 31 to 524 kB of the transcription start site for the proto-oncogenes MECOM, HMGA2, and LMO2. However, none of these clones demonstrated continuity and increasing abundance indicative of insertional mutagenesis, and none were detected in the CD3^+^ enriched postinfection analysis. A majority of the clones were present at only one time point of analysis, and the only detected clone in two longitudinal time points (LMO2 in A11199) decreased in detection frequency from 0.2 to 0.005% of total observed clones. No integrations within 500 kB were detected for either PRDM16 or MN1. In summary, these results indicate that Cal-1 gene marking in our animals is highly polyclonal over 9 months of follow-up in peripheral white blood cells and correlates with total gene marking in peripheral blood.

In addition to monitoring Cal-1 clonality in total peripheral blood prior to SHIV challenge, we enriched CD3^+^ cells from peripheral blood pre- and post-SHIV challenge to determine how infection impacted the clonal distribution within the total T-cell population. Similar to results in total peripheral blood, integration sites in the pre-SHIV challenge (270 days posttransplant) CD3^+^ subset were highly polyclonal. Interestingly, we observed a decrease in the total clonal repertoire detected in this subset after SHIV challenge in each animal. For animal ID A11199, which was challenged with SHIV1157-ipd3N4, this decrease in clonality was consistent with the overall decrease in gene marking in the CD3^+^ subset from 37 to 23% (Figure 7a). By contrast, animal IDs A12309 and A11209 exhibited a decrease in the number of clones, while gene marking increased by approximate two to fourfold (Figure 7b,c). However, the number of clones detected at >1% increased for each animal after infection, suggesting a potential expansion of protected, Cal-1-modified CD3^+^ cells. Together, these data suggest that not only do Cal-1-marked clones persist during SHIV challenge, but more importantly, that these vector-modified cells increase their detected frequency in response to infection while still maintaining a diverse clonal repertoire.

## Discussion

We show that animals transplanted with Cal-1 transduced CD34^+^ cells exhibit long-term, multilineage engraftment of gene-marked cells and that these cells are capable of resisting SHIV challenge *ex vivo* and *in vivo*. Subset sorting experiments reveal a preferential increase in marking in SHIV-susceptible subsets, namely in the T-cell compartment. Marking is also observed in GI tissue, and increased in all three animals over the course of SHIV infection. Finally, our analyses of Cal-1 integration sites not only reinforces the safety of our gene therapy strategy, but also can be utilized to track protected cell clones during ongoing viral replication, and suggest the polyclonal increase of protected cells.

We observed robust Cal-1 gene marking in the CD34^+^ infusion product of each animal and in the bone marrow compartment prior to SHIV challenge. These findings correlated strongly with the long-term, multilineage engraftment of gene-marked cells in the peripheral blood of each animal prior to SHIV challenge. Following challenge, and consistent with our past results,^23^ mC46-marked cells were enriched in a SHIV-dependent manner, leading to progressive decreases in plasma viral load and increases in peripheral blood CD4^+^ T-cell counts over time. Importantly, this clinical-grade vector also expresses a human CCR5-specific short hairpin RNA (“sh5”) that, while not functional in macaque cells, has been shown to efficiently suppress CCR5 expression in humans.^31^ As such, Cal-1 acted as a monotherapy in our model. A strong prediction from our data is that Cal-1 should provide even more robust protection in HIV^+^ patients, where virus entry will be inhibited by both mC46-depdendent and sh5-dependent mechanisms. We are currently evaluating this hypothesis in multiple ongoing/upcoming clinical trials (ClinicalTrials.gov ID NCT01734850 and NCT02378922). More broadly, these results underline the potential contribution of mC46 in the context of multifaceted anti-HIV therapies, which have the greatest potential to induce functional cure.^4^ Our gene therapy-based interventions could be combined with other methods, such as pharmacological anti-latency strategies^44^ or therapeutic vaccination.^45^ Further, by targeting a multicistronic vector such as Cal-1 to a safe harbor locus such as the Adenovirus-Associated Virus Integration Site 1, the safety of our approach could be enhanced.^46^ Independent of the specific methodology, our findings support a key role for mC46 in future combinatorial anti-HIV therapy designs.

Previously, we performed autologous transplants using macaque HSCs that were transduced with a lentiviral vector expressing mC46 and the chemoselection marker MGMT^P140K^.^23,27^ Following hematopoietic recovery, these animals were treated with O^6^-benzylguanine and either *N*,*N*′-bis(2-chloroethyl)-*N*-nitroso-urea (BCNU) or temozolomide to enrich for cells expressing the transgenic selection marker. Interestingly, we observed comparable gene marking and SHIV-dependent positive selection in Cal-1-transplanted animals in relation to our previous results in chemoselected animals. These results suggest that the Cal-1 vector itself is an especially robust method of lentivirus-based gene marking. Although our MGMT^P140K^-dependent chemoselection strategy is safe, the use of Cal-1 avails a strategy to effectively protect cells *in vivo* in contexts where additional administration of chemotherapies may be contraindicated. An important caveat in this study is that our historical controls were not transplanted; the transplant procedure itself may contribute positively or negatively to virus persistence.^47^ Our ongoing studies suggest that maximizing posttransplant recovery time frames will minimize complications attributable to transplant-dependent immune suppression (Peterson *et al*.^47^).

Our data indicate that gene-marked CD4^+^ cells undergo SHIV-dependent positive selection, but CD14^+^ cells do not. Similarly, SHIV-dependent positive selection was modest in total peripheral blood from our animals. These findings suggest that only infection-susceptible cells (those that permit virus entry, *i.e.* CD4^+^CCR5^+^ and/or CD4^+^CXCR4^+^ subsets) undergo virus-mediated positive selection, which is more difficult to measure in a mixed population of susceptible and nonsusceptible cells. Although CD14^+^ cells can be infected, the lack of selection for Cal-1-marked cells in this subset is consistent with the limited proportions that express sufficient levels of CD4 and CCR5/CXCR4 to permit virus entry.^48,49^ The selective upregulation of mC46 in infection-susceptible cells highlights a potential use of Cal-1 to better understand viral pathogenesis in our SHIV-infection model. Our data predict that the infection-susceptible subset of CD14^+^ cells could be identified based on those with an increasing frequency of mC46 protein expression at the cell surface over time following SHIV challenge. We are currently investigating the use of mC46 as a marker capable of revealing rare, infection-susceptible, cellular subsets that may contribute to the persistence of the cART-suppressed viral reservoir.

A second example of the ability of Cal-1 to protect against infection and also inform mechanisms of pathogenesis arises from the ability to track Cal-1 gene-marked cells over time following transplant and virus challenge. By assigning identity to each gene-marked cell on the basis of its unique genomic integration site, this technique has already been exploited by our group and others to better understand the safety and kinetics of hematopoietic reconstitution following stem cell transplant; we have previously demonstrated the utility of our platform in preclinical macaque models as well as in clinical trials.^28,29^ In the context of HIV infection, we demonstrate that Cal-1 gene-marked cells in our SHIV/macaque model display polyclonal engraftment, meaning that a dominant and potentially oncogenic clone was not observed during the course of study (here, at least 20 months). Additionally, no clones with integration sites in proximity (within 500 kB) to several proto-oncogenes previously identified in gene therapy clinical trials were detected with increasing frequency. We further applied this method to address how protected cells respond to virus challenge. In all three animals, we observed a restriction of the detectable clonal repertoire in CD3^+^ cells, which was independent of changes in the overall gene marking of this subset. Despite this restriction, we observed an increase in the number of clones representing greater than 1% of detected sequences, suggesting a potential expansion and increased survival of protected, gene-modified T-cells. This expansion correlates with the dramatic increase in gene marking in two of the animals (A12309 and A11209) challenged with the virulent SHIV strain 89.6P. Additionally, the majority of new clones above the 1% threshold were clones that either had not been previously detected, or were minor contributors suggesting that these cells are protected and persist in response to SHIV challenge. Alternatively, this decrease in detection of total unique clones could be attributed to the loss of cells which either had low or silenced expression of Cal-1, or from nonspecific cell death due to bystander effects, as observed previously by our group and others.^50,51^ Animal IDs A12309 and A11209 exhibited a decrease in unique clones at between 3 and 4 months postchallenge. Interestingly, these animals’ peripheral blood CD4^+^ T-cell counts have gradually increased since this time point was analyzed and may suggest an association between CD4^+^ T-cell rebound and the higher number of clones detected. More broadly, we continue to expand our clonal tracking methodology to better understand the trafficking, survival, and expansion of these subsets,^50^ and also to directly track clonal SHIV integrants, as has been described in the clinical literature.^52,53^

In summary, our data are consistent with a model in which Cal-1-transduced CD34^+^ HSCs, following infusion into conditioned animals, give rise to gene-marked cells in all hematopoietic lineages. In SHIV-susceptible lineages, namely CD4^+^ T-cells, SHIV-dependent selective pressure increases the proportion of gene-marked cells, a phenomenon that we observe both in peripheral blood and in GI tract. This increase in gene-marking correlates with the observed expansion of clones in a SHIV-dependent manner, and this clonal distribution is readily measurable by our integration site methodology. These findings are presently being evaluated in clinical trials, and should also be incorporated into experimental combination therapy approaches aimed at HIV eradication and/or remission.

## Methods

### Cal-1 vector production

Large-scale production of Cal-1 (LVsh5/C46) vector was undertaken by Indiana University Vector Production Facility (IU VPF) as described previously.^54^ The virus-containing media, containing self-inactivating lentiviral vector pseudotyped with vesicular stomatitis virus glycoprotein, was purified by Mustang Q followed by concentration using tangential flow filtration filters. This batch of Cal-1 vector has been thoroughly characterized in previous preclinical studies both *in vitro* and *in vivo* using humanized mouse model systems to assess the safety and efficacy of delivering Cal-1-modified cells to inhibit HIV-1 infection.^20,55^

### Autologous transplant

Three male juvenile pigtailed macaques were utilized in this study and were transplanted consistent with our previously published protocols.^32^ Briefly, animals were primed with granulocyte colony stimulating factor and stem cell factor for 4 days prior to bone marrow harvest. CD34^+^ HSCs were enriched from primed bone marrow and cultured overnight in IMDM + 10% fetal bovine serum, 1% penicillin/streptomycin, and 100 ng/µl each recombinant human stem cell factor, thrombopoietin, and FMS-like tyrosine kinase 3 ligand. Cells were then transduced at a multiplicity of infection of 5 overnight. The following morning, cells were harvested, counted, resuspended to 5 × 10^6^ cells/ml, and pulsed for 2 hours in 10 µM prostaglandin E2 on ice. Finally, cells were resuspended in Hank’s Balanced Salt Solution containing 2% autologous serum and infused into the animal. During the 48-hour *ex vivo* culture period and prior to cell infusion, animals received a fractionated dose of 1,020 cGy total body irradiation. Posttransplant hematopoietic recovery was monitored by automated differential cell counting of multiple cell types, including total white blood cells, platelets, neutrophils, and lymphocytes. We defined *steady-state gene marking* as the average percent gene marking upon stabilization of posttransplant platelet levels (Figure 2b). “Stabilization” was defined as a stretch of 8 consecutive weeks during which platelet counts varied by less than 10% or were above a “healthy minimum” value of 2.6 × 10^5^ platelets/µl whole blood, in the absence of granulocyte colony stimulating factor or blood transfusion support. Animals ID A11199 and A11209 were stabilized at 67 days posttransplant, while animal ID A12309 was stabilized at day 63 (Supplementary Figure S1).

### SHIV challenge and peripheral blood analyses

Intravenous SHIV challenge was conducted at least 200 days following autologous transplant, using challenge stocks identical to those for which historical data is referenced.^36,38^ Blood draws were collected by venipuncture into heparin or EDTA collection tubes. Viral load and CD4^+^ T-cell counts were measured at baseline (pre-SHIV), and 1–4 times per month following challenge, as described previously.^56,57^ “Normal” CD4^+^ T-cell counts were calculated by averaging 2–5 weekly measurements from 26 healthy, SHIV-naive animals, and displayed as a range ±1 SD (Figure 4b). Total leukocytes were collected by hemolysis of whole blood and peripheral blood mononuclear cells (PBMC) were collected by ficoll gradient centrifugation. Total genomic DNA was prepared from each source by Blood Mini Kit (Qiagen, Hilden, Germany) or Masterpure DNA Purification Kit (Epicentre Biotechnologies, Madison, WI). Cal-1 gene marking from total genomic DNA samples was measured by Taqman as described previously.^27^

### *Ex vivo* SHIV assay

Resistance of cultured CD4^+^ cells to infection by SHIV was performed based on our previously published methods.^22^ Prior to SHIV infection, PBMC were isolated via ficoll density gradient separation from each Cal-1-transplanted animal, and from an uninfected, untransplanted control animal. PBMCs were cryopreserved in 90% fetal bovine serum and 10% dimethyl sulfoxide and stored on liquid nitrogen. After all samples were collected, PBMCs were thawed rapidly at 37 °C and diluted into RPMI Media 1640 (Thermo Fisher) with 10% fetal bovine serum , and 1% Pen/Strep antibiotic (RPMI 10/1) with 2 µl of Benzonase nuclease (Novagen) per ml media. Thawed PBMCs were rested overnight in RPMI 10/1 at 37 °C and then enriched for CD4^+^ cells using NHP CD4 Microbeads (Miltenyi Biotec, Bergisch Gladbach, Germany). The enriched CD4^+^ cells were then stimulated with three beads per cell of anti-CD3/CD28 beads in RPMI 10/1 with 2 µl of 100 U/ml IL-2 (Chiron, Emeryville, CA) per 1 ml of RPMI 10/1. Following one day of stimulation and expansion with beads, cells were plated at a density of 6 × 10^4^ cells per well in 48-well non-tissue culture-treated microplates and challenged with SHIV-KU1 in triplicate at multiplicities of infection of 0 and 0.05. Seven days following challenge with SHIV-KU1, cells were harvested and counted for total number of viable cells. DNA was extracted from pooled triplicate samples corresponding to each multiplicity of infection and analyzed for lentivirus gene marking levels by Taqman.

### Colony forming assays

One day following electroporation, 3 × 10^3^ cells were plated in 1-ml methocult media (StemCell Technologies, Vancouver, BC) containing 100 ng/ml each recombinant human IL-3, IL-6, stem cell factor, TPO, granulocyte colony stimulating factor, granulocyte/macrophage colony stimulating factor, and 4 U/ml erythropoietin. Triplicate plates were cultured for up to 2 weeks, until colonies could be visualized. Eight colonies from mock-transduced conditions, and 88 single colonies from Cal-1-transduced conditions were picked into 50-µl QuickExtract DNA Extraction Solution (Epicentre Biotechnologies, Madison, WI) in 96-well plates. DNA was extracted from colonies using the following program: 65 C for 20 minutes, 98 °C for 10 minutes, 4 °C hold, storage at −20 or −80 °C. Extracted colonies were analyzed for Cal-1 marking by gel PCR using primers against lentiviral backbone (F: AGAGATGGGTGCGAGAGCGTCA, R: TGCCTTGGTGGGTGCTACTCCTAA) and actin (F: TCCTGTGGCACTCACGAAACT, R: GAAGCATTTGCGGTGGACGAT). Analagous methods were applied to hemolysed bone marrow white blood cell samples collected from each animal immediately prior to (200–300 days posttransplant) and following SHIV challenge (300–400 days posttransplant, 100 days postinfection).

### Subset sorting

Following hematopoietic recovery, and prior to (200–300 days posttransplant) or following SHIV challenge (300–400 days posttransplant, 100 days postinfection), large volume blood draws were collected by venipuncture into heparin collection tubes. Whole blood was separated by Ficoll centrifugation, and the mononuclear cell layer was subjected to bead-based positive selection with CD3, CD4, or CD14 beads (Miltenyi Biotec, Bergisch Gladbach, Germany). Subset purities were confirmed by flow cytometry, and the remainder of cells were processed for total genomic DNA for analysis of Cal-1 gene marking by Taqman.

### GI biopsies

Upper and lower GI biopsies were collected and processed as described previously.^38^ Importantly, although all efforts were made to minimize contamination of these samples with peripheral blood, it is impossible to exclude the possibility that low levels of circulating leukocytes were collected during these survival surgeries. Total genomic DNA was isolated from dissociated cells as described above, and Cal-1 gene marking was measured by Taqman.

### Integration site analysis

Retroviral integration site analysis was performed using our modified genome sequencing-PCR protocol as previously described.^28,29^ Briefly, DNA was extracted from hemolysed peripheral blood as described above, and 3 µg was randomly sheared using an M220 focused ultrasonicator (Covaris). Fragmented DNA was purified, polished (End-It DNA End Repair Kit, Epicentre) and ligated to modified linker cassettes containing known primer binding sites. This product was then amplified using sequential nested exponential PCR reactions (primer pair 1, longer terminal repeat-specific, L2-PST-1: 5′-biotin-AGCTTGCCTTGAGTGCTTCA-3′ and linker-specific LC1: 5′-GACCCGGGAGATCTGAATTC-3′; primer pair 2, longer terminal repeat-specific L2-(Barcode)-2A: 5′-CCATCTCATCCC TGCGTGTCTCCGACTCAG-(Barcode)-AGTAGTGTGTGCCCGTCTGT-3′ and linker-specific LC2-trP1: 5′-CTA TGCGCCTTGCCAGCCCGCTCAGGATCTGAATTCAGTGGCACAG-3′). Product from the first PCR was captured using biotin specific beads (Life Technologies), and eluted DNA was diluted prior to the second nested PCR, which added both barcodes and sequences required for compatibility with massively paralleled semiconductor sequencing (IonTorrent, Life Technologies). PCR products were visualized on 2% agarose gels, and DNA fragments ranging between 300–800 bp were extracted, purified, and sequenced by Edge Biosciences (Gaithersburg, MD). Processing and genomic mapping of integration sites was carried out as previously described^28^ with the following exceptions: valid integration sites were scored after locating the retroviral longer terminal repeat-gDNA junction and liker cassette sequences. The resulting gDNA sequences were aligned to the October 2010 (BGI CR_1.0/rheMac3) assembly of the rhesus genome using the UCSC Genome Browser, which generates a BLAST alignment score. To analyze integrations near proto-oncogenes, genes previously associated with oncogenic transformation in clinical trials (LMO2, MECOM, PRDM16, HMGA2, and MN1) were converted from human (hg38) genomic coordinates to the corresponding rhesus locus using the same rheMac3 genome browser. A custom python script was created to map identified integration sites to the nearest genomic transcription start site and identified sites which mapped near or within the previously mentioned genes of interest. The clonal frequency of these insertion sites was tracked over time for continuity and abundance.

### Statistical analyses

Statistically significant differences in lentiviral gene marking and *ex vivo* proliferation of SHIV-challenged T-cells were determined using one-tailed Student’s *t*-tests with unequal sample sizes and unequal variance. For tests of significance from colony forming experiments, a *z*-statistic was calculated prior to generation of *P* values.

### Ethics statement

This study was carried out in strict accordance with the recommendations in the Guide for the Care and Use of Laboratory Animals of the National Institutes of Health (“The Guide”). The protocol was approved by the Institutional Animal Care and Use Committees of the Fred Hutchinson Cancer Research Center and University of Washington, Protocol # 3235-01. All animals were housed at and included in standard monitoring procedures prescribed by the Washington National Primate Research Center (WaNPRC). This included at least twice-daily observation by animal technicians for basic husbandry parameters (*e.g.* food intake, activity, stool consistency, and overall appearance) as well as daily observation by a veterinary technician and/or veterinarian. Animals were housed in cages approved by “The Guide” and in accordance with Animal Welfare Act regulations. Animals were fed twice daily, and were fasted for up to 14 hours prior to sedation. Environmental enrichment included grouping in compound, large activity, or run-through connected cages, perches, toys, food treats, and foraging activities. If a clinical abnormality was noted by WaNPRC personnel, standard WaNPRC procedures were followed to notify the veterinary staff for evaluation and determination for admission as a clinical case. In particular, simian AIDS clinical criteria included >15% loss of baseline body weight, sustained hematocrit <15%, CD4^+^ T-cell count <200/µl, and presence of unresponsive opportunistic infection or other clinical condition. Animals were sedated by administration of ketamine HCl and/or telazol and supportive agents prior to all procedures. Following sedation, animals were monitored according to WaNPRC standard protocols. WaNPRC surgical support staff are trained and experienced in the administration of anesthetics and have monitoring equipment available to assist: electronic monitoring of heart rate, respiration, and blood oxygenation; audible alarms and LCD readouts; monitoring of blood pressure, temperature, etc. For minor procedures, the presence or absence of deep pain was tested by the toe-pinch reflex. The absence of response (leg flexion) to this test indicates adequate anesthesia for this procedure. Similar parameters were used in cases of general anesthesia, including the loss of palpebral reflexes (eye blink). Analgesics were provided as prescribed by the Clinical Veterinary staff for at least 48 hours after the procedures, and could be extended at the discretion of the clinical veterinarian, based on clinical signs. Decisions to euthanize animals were made in close consultation with veterinary staff and were performed in accordance with guidelines as established by the American Veterinary Medical Association Panel on Euthanasia (2013). Prior to euthanasia, animals were first rendered unconscious by administration of ketamine HCl.
