# Supplementary material for: Multilineage polyclonal engraftment of Cal-1 gene-modified cells and in vivo selection after SHIV infection in a nonhuman primate model of AIDS
Source: Mol Ther Methods Clin Dev. 2016 Feb 24;3:16007–. doi: 10.1038/mtm.2016.7 (PMC4765711; doi:10.1038/mtm.2016.7)
Supplement: Supplementary Figure and Table [file mtm20167-s1.docx]

**SUPPLEMENTAL DATA FILE for**

**MS# MTM-00265-T: Multilineage polyclonal engraftment of Cal-1 gene-modified cells and in vivo selection after SHIV infection in a Nonhuman Primate Model of AIDS**

**Two Supplemental Items: Supplemental Figure S1 and Supplemental Table S1**

**Supplemental Figure S1
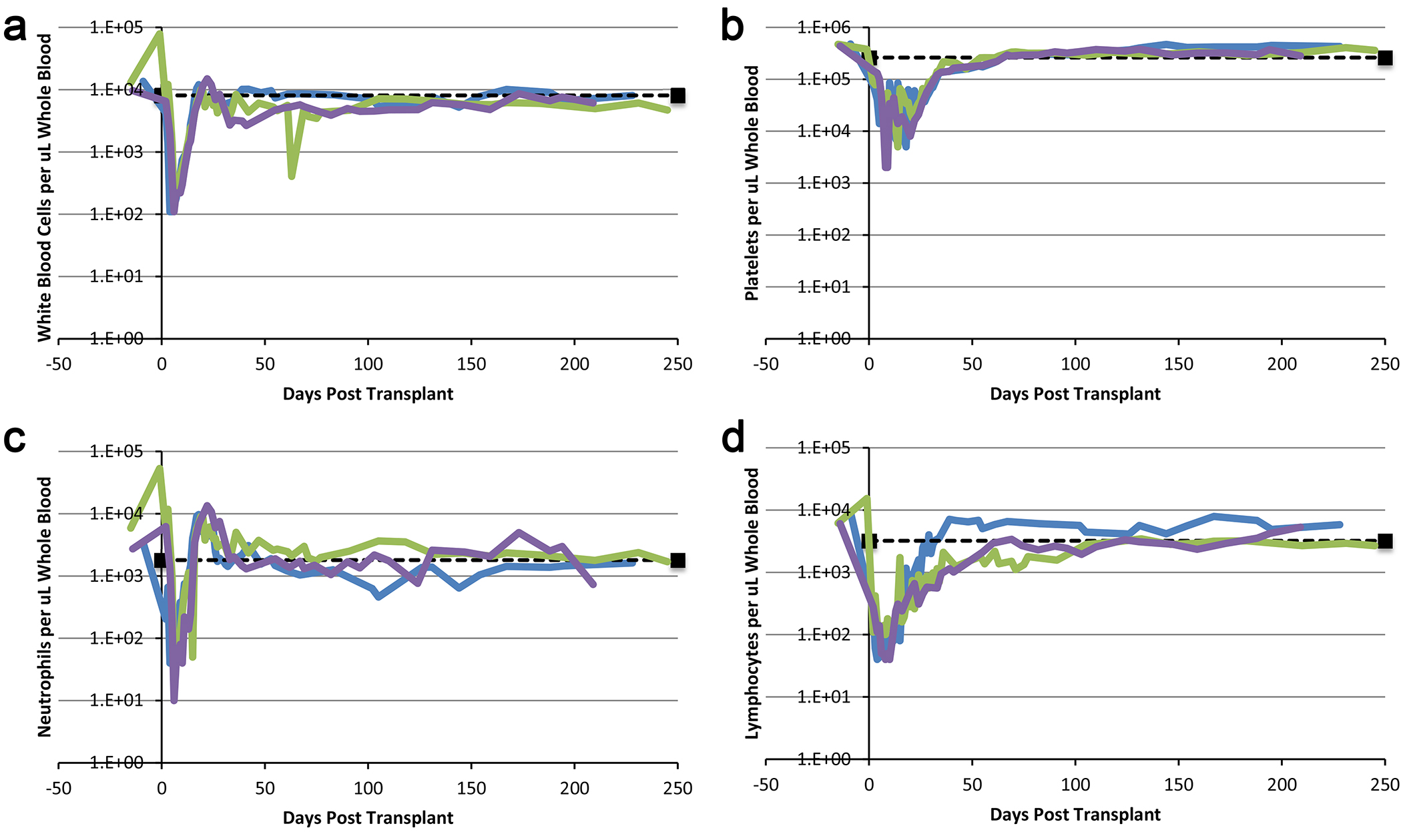
**

**Supplemental Figure S1. Hematopoietic reconstitution in three pigtailed macaques following autologous transplant with Cal-1-transduced CD34+ cells.** At the indicated time points post-transplant, whole blood samples were counted for total white blood cells (**a**), platelets (**b**), neutrophils (**c**), and lymphocytes (**d**) by automated differential blood count. Counts are expressed as cells of interest per µl whole blood. Dashed lines indicate expected counts in a normal animal. Blue: Animal ID A11199. Green: Animal ID A12309. Purple: Animal ID A11209

**Supplemental Table T1**

**Supplemental Table S1. Summary of detected clones containing integration sites near known proto-oncogenes.** Integration sites in proximity to the indicated gene of interest for each individual animal are shown including the distance to the transcription start site of the gene, the relative frequency of detection (1.29E-03 is equivalent to 0.129% of total clones detected at that time), and whether the insertion was within the gene coding region. Identical clones detected at multiple timepoints are indicated with symbols (* ^# +^).
